# *CCR2* and *CCR5* genes polymorphisms in women with cervical lesions from Pernambuco, Northeast Region of Brazil: a case-control study

**DOI:** 10.1590/0074-02760150367

**Published:** 2016-03

**Authors:** Erinaldo Ubirajara Damasceno dos Santos, Géssica Dayane Cordeiro de Lima, Micheline de Lucena Oliveira, Sandra de Andrade Heráclio, Hildson Dornelas Angelo da Silva, Sergio Crovella, Maria de Mascena Diniz Maia, Paulo Roberto Eleutério de Souza/

**Affiliations:** 1 Universidade Federal Rural de Pernambuco, Programa de Pós-Graduação em Ciência Animal Tropical, Recife, PE, Brasil; 2 Universidade de Pernambuco, Programa de Pós-Graduação em Biologia Celular e Molecular Aplicada, Recife, PE, Brasil; 3 Laboratório Central de Saúde Pública de Pernambuco, Recife, PE, Brasil; 4 Instituto de Medicina Integral Prof. Fernando Figueira, Recife, PE, Brasil; 5 Instituto Federal de Pernambuco, Garanhuns, PE, Brasil; 6 Universidade Federal de Pernambuco, Programa de Pós-Graduação em Genética, Recife, PE, Brasil

**Keywords:** chemokine receptors, cervical intraepithelial neoplasia, cervical cancer, single nucleotide polymorphism

## Abstract

Polymorphisms in chemokine receptors play an important role in the progression of cervical intraepithelial neoplasia (CIN) to cervical cancer (CC). Our study examined the association of *CCR2-64I* (rs1799864) and*CCR5-Δ32* (rs333) polymorphisms with susceptibility to develop cervical lesion (CIN and CC) in a Brazilian population. The genotyping of 139 women with cervical lesions and 151 women without cervical lesions for the *CCR2-64I* and *CCR5-Δ32* polymorphisms were performed using polymerase chain reaction-restriction fragment length polymorphism. The individuals carrying heterozygous or homozygous genotypes (GA+AA) for *CCR2-64I* polymorphisms seem to be at lower risk for cervical lesion [odds ratio (OR) = 0.37, p = 0.0008)]. The same was observed for the A allele (OR = 0.39, p = 0.0002), while no association was detected (p > 0.05) with *CCR5-Δ32* polymorphism. Regarding the human papillomavirus (HPV) type, patients carrying the *CCR2-64I*polymorphism were protected against infection by HPV type 16 (OR = 0.35, p = 0.0184). In summary, our study showed a protective effect of*CCR2-64I* rs1799864 polymorphism against the development of cervical lesions (CIN and CC) and in the susceptibility of HPV 16 infection.

Infections by oncogenic types of human papillomavirus (HPV) are found in 99% of women with cervical cancer (CC) (de Oliveira et al. 2013). Therefore, this virus is classified as the most important carcinogenic risk factor according to the criteria of the International Agency for Research on Cancer (Bonanni et al. 2015). Among more than 120 types of HPV have been identified, 18 of these are classified as high-risk oncogenic (Bouvard et al. 2009, Bernard et al. 2010). However, the majority of women infected with HPV will not develop cervical carcinoma, since the carcinogenic process depends on several other genetic, environmental, and immune factors (Zheng et al. 2006).

The loss of cell cycle control mechanism, infiltration of leukocyte, and of other immunocompetent cells, as well as the altered expression of immune response genes, have been considered critical in neoplasms cervical pathogenesis and progression for CC (Evans et al. 1997, Ghaderi et al. 2000, O’Brien et al. 2001).

Among the gene products present into cervical mucosa, chemokines and their receptor have shown a key role in immunity against cervical tumours (Ghaderi et al. 2000, Ohta et al. 2002). Chemokines are chemoattractant proteins of low molecular weight that promote adhesiveness of target cells; then the angiogenesis process drive homing of phagocytes and lymphocytes into secondary lymphoid organs (Rossi & Zlotnik 2000, Kleine-Lowinski et al. 2003, Zheng et al. 2006). The receptors for chemokines are mainly expressed on immune cells and assist in the differentiation and migration of these cells to inflamed tissues (Bromley et al. 2005).

Chemokine receptor (CCR)5 is the major receptor for the chemokine and their ligands are the macrophage inflammatory protein (MIP)-1α/chemokine ligand (CCL)3, MIP-1β/CCL4, and regulated on activation, normal T cell expressed and secreted/CCL5 (Lehner 2002, Al-Abdulhadi & Al-Rabia 2010, Ahmadabadi et al. 2012). Polymorphic variations in this gene, in particular the Δ32 mutation (a 32 bp deletion in the *CCR5* gene) leads to decreased expression and dysfunction of CCR5 receptor (Nahon et al. 2008, Ahmadabadi et al. 2012). Studies report that individuals homozygotes for *CCR5-Δ32* (rs333) gene have reduced risk for asthma and early-onset myocardial infarction, attenuation of severity in rheumatoid arthritis, and slower acquired immune deficiency syndrome (AIDS) progression (Berger et al. 1999, Hall et al. 1999, Zapico et al. 2000, González et al. 2001). In addition CCR5, together with CCR2, act as co-receptors for human immunodeficiency virus-1 (Zheng et al. 2006).

CCR 2 is the receptor for CCL 2, also known as monocyte chemoattractant protein (MCP)-1, being associated with carcinogenesis and angiogenesis (Charo et al. 1994, Zhang et al. 2003,Koide et al. 2004, Huang et al. 2013). The single nucleotide polymorphism at codon 64 (*CCR2-64I*, rs1799864) of *CCR2* gene that encodes isoleucine (ATC) instead of valine (GTC) has been widely studied, and there are reports of association between this polymorphism and the protective effect in the progression of inflammatory diseases such as multiple sclerosis (Miyagishi et al. 2003), carotid atherosclerosis (Nyquist et al. 2009), and in development of breast cancer (Zafiropoulos et al. 2004). However, conflicting results on the role of the *CCR5* and *CCR2* polymorphisms in the development of the CC have been reported so far (Coelho et al. 2005, Zheng et al. 2006, Ivansson et al. 2007, Chatterjee et al. 2010).

Therefore, the aim of this study was to analyse the association for*CCR2-64I* and *CCR5-Δ32* polymorphisms with development of cervical intraepithelial neoplasia (CIN) or CC in women infected by HPV from Northeast Region of Brazil.

## SUBJECTS, MATERIALS AND METHODS

*Population* - The present study was a hospital-based cross-sectional prospective one carried out in the outpatient clinics of the Lower Genital Tract Pathology Clinic at the Women’s Healthcare Center of the Prof Fernando Figueira Institute of Integrated Medicine, Recife, state of Pernambuco, Brazil. Patients were selected by spontaneous demand from January 2009 until 2011 and the study population consisted of 290 sexually active women ranging between 16-75 years old. Information was collected from all women pertaining to their age, smoking, alcohol consumption, number of offspring, number of sexual partners, and age at first coitus. The inclusion criteria was as follows: women with oncotic cytology submitted to Papanicolaou test (cytological) according to Bethesda System terminology (Solomon et al. 2002) performed on the state accredited networks, presenting diagnostic of CIN of low-grade and high-grade or CC, and confirmed by histological analysis. Subjects were evaluated for clinical features of other sexually transmitted infections on history and examination. Patients that were previously submitted to radiotherapy or chemotherapy to invasive CC were excluded. The Institutional Ethical Committee approved this study (protocol 355/08). Informed written consent was taken from the women informing them about the background of the study, risks and benefits, and voluntary nature of participation. After histological analysis, patients were stratified according to the presence or absence of cervical lesion (CIN or CC) as case and control groups, respectively.

*Clinical samples -* Cervical smears were obtained using Cytobrushes. Each Cytobrush was packed in a Tris-ethylenediamine tetraacetic acid (EDTA) buffer solution (Tris-HCl 10 mM and EDTA 1 mM pH 8.0) and conserved at -20ºC until analysis.

*DNA extraction* - Genomic DNA extraction was performed from 300 µL of vaginal fluid from each study subject, following the manufacturer’s instructions of the kit Wizard^®^ Genomic DNA Purification (Promega, USA). The analyses samples were executed the Laboratory of Genetics, Biochemistry, and DNA Sequencing at Rural Federal University of Pernambuco.

*HPV detection and typing* - Amplification of human β-globin gene segment was used as an internal control for DNA quality and samples negative for this assay were excluded from analysis. Then, our samples were tested for HPV presence using MY09/11, GP05+ and GP06+ consensus primers by polymerase chain reaction (PCR) (Tavares et al. 2014). The typing of high-risk HPV (HR-HPV) 16, 18, 31, and 33 was performed using specific primers (da Silva et al. 2009, Tavares et al. 2015).

*Analysis of the CCR2-64I polymorphism -* The*CCR2-64I* polymorphism was analysed through PCR followed by restriction fragment length polymorphism (Coelho et al. 2005). DNA was amplified using primers sense 5’-TTGTGGGCAACATGATGG-3’ and antisense 5’-GCATTCCCAAAGACCCACTC-3’. The PCR products of 163 bp length were then digested with *BsaBI* restriction enzyme. The fragments originated after of the use the restriction enzyme, 163 bp for G allele, and 145 and 18 bp for A allele were revealed using 3% agarose gel stained with gel red (UNISCIENCE).

*Analysis of the CCR5-Δ32 polymorphism* - The*CCR5-Δ32* polymorphism was analysed through PCR (Kristiansen et al. 2001) using primers sense 5’-CTTCATCATCCTC-CTGACAATCG-3’ and antisense 5’-GACCAGCCCCAAGTTGACTATC-3’. PCR products of 262 bp for CCR5 wild-type allele and 230 bp for*CCR5-Δ32* allele were detected with 3% agarose gel stained with gel red (UNISCIENCE).

*Sequencing* - A total of 20% of the all samples (randomly chosen) was submitted to bidirectional sequencing (MegaBACE 1000 DNA sequencer; GE Healthcare, USA) in order to double-check the genotyping results for each polymorphism (Tavares et al. 2015).

*Statistical analyses* - Univariate statistical analysis was performed using the BioEstat 5.0 software. The study was cross-sectional with independent samples consisting of nominal data (genotypes). The influence of each polymorphism on the risk for development of (pre) neoplastic cervical disease was estimated by odds ratio (OR) and a 95% confidence interval (CI). Allele frequencies were estimated by direct counting. Comparison between genotypic frequencies of patients and control groups was performed by chi-square test and Fisher’s exact test was used to compare the allele frequencies in contingency tables.

For identification of relevant risk factors, a logistic regression analysis was carried out (comparing HPV-positive women with history of lesions with HPV-positive women with no history of lesions or CC). This modelled the influence of genetic polymorphisms, HPV 16 single infection or multiple HPV strains co-infection, smoking and alcohol consumption on the risk of developing high-grade squamous intraepithelial lesions. The OR and their respective 95% CI were determined. The R software v.3.0.2 (R-project.org/) was used to perform the regression analysis. Power analysis was performed through G*Power software v.3.1.9.2 (Faul et al. 2007). All p-values ≤ 0.05 were considered statistically significant.

## RESULTS

Within the 290 HPV positive enrolled women, 139 had cervical lesions (CIN or CC) (HPV+L) while the 151 had no cervical lesions (HPV+). When considering the prevalence of type-specific HR-HPV infection, we observed that 38.8% of the patients had HPV 16, 22.3% HPV 18, 2.9% HPV 31, 3.6% HPV 33, and 14.4% other HPV types. Furthermore, the presence of co-infection by HPV types 16/18 was found in 18% of the patients (Table I). Moreover, when the patients were stratified according to the severity of cervical lesions, 28.78% (40/139) exhibited CIN I (low-degree of lesion), 62.58% (87/139) had CIN II or III (high-degree of lesion), and 8.63% (12/139) had CC.

**TABLE I:** Human papillomavirus (HPV) genotypes prevalence and histologic diagnosis

Types	CIN I (n = 40) n (%)	CIN II/III (n = 87) n (%)	CC (n = 12) n (%)	Total (n = 139) n (%)
HPV 16	10 (25)	38 (43.7)	6 (50)	54 (38.8)
HPV 18	9 (22.5)	20 (22.9)	2 (16.7)	31 (22.3)
HPV 31	2 (5)	2 (2.3)	0 (0)	4 (2.9)
HPV 33	0 (0)	4 (4.6)	1 (8.3)	5 (3.6)
Others HPV	14 (35)	5 (5.8)	1(8.3)	20 (14.4)
Co-infection (16/18)	5 (12.5)	18 (20.7)	2 (16.7)	25 (18)

CC: cervical cancer; CIN: cervical intraepithelial neoplasia.

Among the 139 HPV+L, five risk factors for cervical lesions were analysed: smoking, alcohol consumption, number of offspring, number of sexual partners, and age at first coitus. With the 139 HPV+L, 32.37% (45/139) reported smoking, 60.03% (89/139) alcohol consumption, 28.78% (40/139) number of offspring > 3, 20.17% (24/119) had number of sexual partners > 4, and 59.71% (83/139) had first coitus with ≤ 16 years old.

The distribution of *CCR2-64I* and *CCR5-Δ32*polymorphisms genotypes in 139 women with cervical lesions (CIN or CC) and 151 HPV+ were according to the Hardy-Weinberg equilibrium. A significant difference in the distribution of *CCR2-64I* polymorphism between HPV+ L and HPV+ was observed using a dominant genetic model (OR = 0.37; p = 0.0005), being the variant carrier (GA+AA) associated with protection to cervical lesions (Table II). When considering the*CCR5-Δ32* polymorphism, no statistical difference between HPV+-L and HPV+ was observed (p = 0.3943) (Table II).

**TABLE II:** Genotypic distribution of the *CCR2-64I* and*CCR5-Δ32* gene polymorphisms in human papillomavirus (HPV) positive patients with cervical intraepithelial neoplasia (CIN) and cervical cancer (CC) (HPV+L) or without cervical lesions (HPV+)

SNP	HPV+ (n = 151) n (%)	HPV+L (n = 139) n (%)	χ2 (p)	OR (95% CI)	p^*a*^
*CCR2 64I* genotypes					
GG	99 (65.6)	116 (83.4)		1	
GA	43 (28.4)	21 (15.1)	8.820 (0.0047)	**0.42 (0.23-0.75)**	**0.0047**
AA	9 (6)	2 (1.5)	5.367 (0.0447)	**0.19 (0.04-0.89)**	**0.0447**
AA + GA x GG	52/99 (52.5)	23/116 (19.8)	12.082 (0.0005)	**0.37 (0.21-0.66)**	**0.0008**
Allele					
G	241 (79.8)	253 (91)	14.393 (0.0001)	1	
A	61 (20.2)	25 (9)		**0.39 (0.23-0.64)**	**0.0002**
*CCR5-Δ32* genotypes					
WT/WT	141 (93.3)	125 (89.9)		1	
WT/Δ32	10 (6.7)	14 (10.1)	1.134 (0.2868)	1.57 (0.67-3.68)	0.3943
Δ32/Δ32	0 (0)	0 (0)	ND	ND	ND
WT/Δ32 + Δ32/Δ32 x WT/WT	10/141 (7)	14/125 (11.2)	1.134 (0.2868)	1.57 (0.67-3.68)	0.3943
Allele					
WT	292 (96.7)	264 (94.9)		1	
Δ32	10 (3.3)	14 (5.1)	1.085 (0.2975)	1.54 (0.67-3.54)	0.4047

*a*: value of the odds ratio (OR); CI: confidence interval; ND: not determined; SNP: single nucleotide polymorphism; WT: wild-type; χ^2^: chi-square test. Bolded values mean significant values.

Statistical significant association was found between *CCR2-64I*polymorphism and susceptibility to HPV 16 infection (OR = 0.35; p = 0.0184) (Table III); the *CCR5-Δ32*variant did not show any association with HPV types studied.

**TABLE III:** Genotypic distribution of the *CCR2-64I* and*CCR5-Δ32* polymorphisms in human papillomavirus (HPV)-16 positive patients and in patients with HPV genotypes other than type 16

SNP	Other HPV (n = 85) n (%)	HPV 16 (n = 54) n (%)	χ2 (p)	OR (95% CI)	p^*a*^
*CCR2 64I* genotypes					
GG	69 (81.1)	47 (87.1)	1		
GA	1 (1.1)	6 (11.1)	13.76 (0.001)	8.80 (1.02-75.56)	0.0509
AA	15 (17.8)	1 (1.8)		**0.09 (0.01-0.77)**	**0.0167**
GG/GA + AA	69/16	47/7		0.64 (0.24-1.68)	0.5015
Allele					
G	139 (81.7)	100 (92.6)	6.93 (0.031)	1	
A	31 (18.3)	8 (7.4)		**0.35 (0.15-0.81)**	**0.0184**
*CCR5-Δ32* genotypes					
WT/WT	80 (94.1)	46 (85.2)		1	
WT/Δ32	5 (5.9)	8 (14.8)	3.01 (0.221)	2.78 (0.86-9.01)	0.1432
Δ32/Δ32	0	0		ND	
WT/WT x WT/Δ32 + Δ32/32	80/5	46/8			
Alelle					
WT	165/170 (97.1)	100/108 (92.6)		1	
Δ32	5/170 (2.9)	8/108 (7.4)	2.86 (0.239)	2.64 (0.84-8.29)	0.1534

*a*: value of the odds ratio (OR); CI: confidence interval; ND: not determined; SNP: single nucleotide polymorphism; WT: wild-type; χ^2^: chi-square test. Bolded values mean significant values.

Patients clinical features are shown in Table IV. There were no significant statistical differences between*CCR2-64I* and *CCR5-Δ32* polymorphisms with age, smoking, alcohol consumption, number of offspring, number of sexual partners, or age at first coitus (p > 0.05).

**TABLE IV:** Genotypic distribution of the *CCR2-64I* and*CCR5-Δ3*2 polymorphisms in patients with cervical lesions (cervical intraepithelial neoplasia and cervical cancer and clinical features

Clinical features	*CCR2-64I*						

	A/P	(n = 139)	G/G	G/A + A/A	χ^2^ (p)	OR (95% CI)	p^a^
Smoking	P	45	36	9	0.4484	1.42 (0.56-3.60)	0.6072
	A	94	80	10			
Alcohol consumption	P	89	77	12	0.1947	0.55 (0.41-4.36)	0.2896
	A	50	39	11			
Number of offspring	> 3	40	33	7	0.7452	1.18 (0.42-3.28)	0.9521
	≤ 3	79	67	12			
Number of sexual partners	> 4	24	21	3	0.6038^*a*^	0.70 (0.18-2.65)^*a*^	0.8360^*a*^
	≤ 4	95	79	16			
Age at first coitus	≤ 16	83	67	16	0.2916	1.67 (0.63-4.37)	0.4111
	≤ 16	56	49	7			

	*CCR5-Δ32*						

Clinical features	A/P	(n = 139)	Wt/Wt	Wt/Δ32 + Δ32/Δ32	χ^2^ (p)	OR (95% CI)	p^*a*^

Smoking	P	45	40	5	0.6222	1.34 (0.41-4.36)	0.8561
	A	94	86	8			
Alcohol consumption	P	89	79	10	0.0529	6.20 (0.77-49.96)	0.1077
	A	50	49	1			
Number of offspring	> 3	40	36	4	0.9827	0.76 (0.27-3.49)	0.7637
	≤ 3	79	71	8			
Number of sexual partners	> 4	24	22	2	0.7499^*a*^	0.77 (0.15 -3.78)^*a*^	0.9517
	≤ 4	95	85	10			
Age at first coitus	≤ 16	83	76	7	0.6506	0.76 (0.24-2.41)	0.8761
	> 16	56	50	6			

*a*: were used a total of 119 patients; A: absence of the characteristic; CI: confidence interval; OR: odds ratio of A x G alleles; P: presence of the characteristic; WT: wild-type; χ2: chi-square test.

## DISCUSSION

In this study, we examined the possible association between *CCR2-64I*and *CCR5-Δ32* polymorphisms and the presence of cervical lesions (CIN or CC) in HPV infected women from Northeast Region of Brazil.

The CCRs genes *CCR5* and *CCR2* have been associated with carcinogenesis and angiogenesis (Zheng et al. 2006), inflammatory disorders, and autoimmune diseases (Rossi & Zlotnik 2000). Immunological studies, regarding to immune response genes, showed a significant decrease in intraepithelial macrophages (Tay et al. 1987), Langerhans cells (Spinillo et al. 1993), and cytotoxic T lymphocytes in CIN advanced (Evans et al. 1997).

The *CCR2-64I* polymorphism has been reported to influence several diseases as multiple sclerosis (Miyagishi et al. 2003), carotid atherosclerosis (Nyquist et al. 2009), breast cancer (Zafiropoulos et al. 2004), AIDS progression (Smith et al. 1997, Mulherin et al. 2003), and CIN or CC (Coelho et al. 2005, Ivansson et al. 2007, Chatterjee et al. 2010). Our results showed a protective effect of *CCR2-64I* polymorphic variant against the development of cervical lesions (OR = 0.37); Coelho et al. (2005)found the same outcome in a Portuguese population. Nevertheless, Chatterjee et al. (2010) and Ivansson et al. (2007) reported that the A allele conferred risk to development of CC in African and Swedish women. The contradictory results on the A allele in relation to the development of CIN or CC (Coelho et al. 2005, Zheng et al. 2006, Ivansson et al. 2007) might be due to its conflicting role in the macrophages recruitment reported by some authors. Wallin et al. (1999) suggested that mutant allele*CCR2-64I* can be linked with decreased macrophages recruitment in the process of tumour angiogenesis, which could be a key during progression of cervical neoplasia to CC. However, Chatterje et al. (2010) proposed that the raised attraction of the macrophages through the increased expression of MCP-1 could be auxiliary in the process of destruction or progression of tumour. These discordant findings reinforce the possibility of several factors associated with the multifactorial neoplastic development, including the difference in ethnic origin of the populations studied, sample sizes, and the low percentage of the mutant allele (*CCR2-64I*).

Studies reported that *CCR5-Δ32* is involved in slower AIDS progression (Berger et al. 1999), in decreasing the severity of rheumatoid arthritis (Zapico et al. 2000), and in the reduced risk to asthma (Hall et al. 1999). To our knowledge until now, only one study conducted by Zheng et al. (2006) in a Swedish population related the *CCR5-Δ32*polymorphism with CC. The authors observed that individual carriers of the allele Δ32 had 4.58 fold-increased risk to HPV infection, but they did not found association in relation to progression of cervical lesion. In our study, we did not observe any association of Δ32 variant with development of cervical lesion (p > 0.05). However, to clarify the role of this genotype in both HPV infection and progression for cervical lesion, the *CCR5-Δ32* polymorphism should be tested in other populations from different ethnic background.

Regarding to prevalence of HPV infection, we found that the more frequent types were HPV 16 and 18 (38.8% and 22.3%, respectively). The prevalence of HPV 31 was 2.8% and HPV 33 was 3.5%. Tavares et al. (2014) found similar results in a study conducted at Recife with 142 HPV positive women with cervical lesion. Silva et al. (2003) andRabelo-Santos et al. (2003) observed that HPV type 16 was the most prevalent virus in all Brazilian regions, but there were variation regarding the frequencies of the type 16 in relation to other types. Furthermore, ours results are in agreement with distribution of these HPV types worldwide in women with CC (Li et al. 2011). da Silva et al. (2009) evaluated the incidence of HPV types in 213 samples cervical of women in Recife, finding a higher frequency of HPV 16 (78%), HPV 31 (15.5%), and lower frequency of HPV 18 (2.8%) when compared to our findings. Lorenzato et al. (2000) also found higher prevalence of HPV 31 (21.4%) and a lower prevalence of HPV 18 (2.4%).

Previous reports have shown that concomitant or sequential detection of more than one HPV type it is associated with different stages of cervical lesions (Silva et al. 2003, da Silva et al. 2009, Tavares et al. 2014). Our results are in agreement with the above mentioned findings: in 25 samples co-infected by HPV 16/18, 80% (20) had high-grade of cervical lesions (neoplasia intraepithelial cervical II/III or CC).

A protector effect of the *CCR2-64I* polymorphism with the susceptibility of infection by HPV type 16 (OR = 0.35) was observed, suggesting a possible relation of this genetic variant with protection to HPV-16 infection.

We also compared clinical features from patients with genotypic distribution of both*CCR2-64I* and *CCR5-Δ32* polymorphisms. However, no significant difference was observed (p > 0.005). The frequencies of*CCR2* and *CCR5* variants alleles were in agreement with a previous study in different diseases and ethnic groups (Lawhorna et al. 2013).

Thus, our data suggest that the *CCR2-64I* polymorphism is associated with the protective effect to development of cervical lesions as well as in the protection to HPV 16 infection.
